# Fibre-Specific Responses to Endurance and Low Volume High Intensity Interval Training: Striking Similarities in Acute and Chronic Adaptation

**DOI:** 10.1371/journal.pone.0098119

**Published:** 2014-06-05

**Authors:** Trisha D. Scribbans, Brittany A. Edgett, Kira Vorobej, Andrew S. Mitchell, Sophie D. Joanisse, Jennifer B. L. Matusiak, Gianni Parise, Joe Quadrilatero, Brendon J. Gurd

**Affiliations:** 1 School of Kinesiology and Health Studies, Queen’s University, Kingston, Ontario, Canada; 2 Department of Kinesiology, University of Waterloo, Waterloo, Ontario, Canada; 3 Department of Kinesiology, McMaster University, Hamilton, Ontario, Canada; West Virginia University School of Medicine, United States of America

## Abstract

The current study involved the completion of two distinct experiments. Experiment 1 compared fibre specific and whole muscle responses to acute bouts of either low-volume high-intensity interval training (LV-HIT) or moderate-intensity continuous endurance exercise (END) in a randomized crossover design. Experiment 2 examined the impact of a six-week training intervention (END or LV-HIT; 4 days/week), on whole body and skeletal muscle fibre specific markers of aerobic and anaerobic capacity. Six recreationally active men (Age: 20.7±3.8 yrs; VO_2_peak: 51.9±5.1 mL/kg/min) reported to the lab on two separate occasions for experiment 1. Following a muscle biopsy taken in a fasted state, participants completed an acute bout of each exercise protocol (LV-HIT: 8, 20-second intervals at ∼170% of VO_2_peak separated by 10 seconds of rest; END: 30 minutes at ∼65% of VO_2_peak), immediately followed by a muscle biopsy. Glycogen content of type I and IIA fibres was significantly (p<0.05) reduced, while p-ACC was significantly increased (p<0.05) following both protocols. Nineteen recreationally active males (n = 16) and females (n = 3) were VO_2_peak-matched and assigned to either the LV-HIT (n = 10; 21±2 yrs) or END (n = 9; 20.7±3.8 yrs) group for experiment 2. After 6 weeks, both training protocols induced comparable increases in aerobic capacity (END: Pre: 48.3±6.0, Mid: 51.8±6.0, Post: 55.0±6.3 mL/kg/min LV-HIT: Pre: 47.9±8.1, Mid: 50.4±7.4, Post: 54.7±7.6 mL/kg/min), fibre-type specific oxidative and glycolytic capacity, glycogen and IMTG stores, and whole-muscle capillary density. Interestingly, only LV-HIT induced greater improvements in anaerobic performance and estimated whole-muscle glycolytic capacity. These results suggest that 30 minutes of END exercise at ∼65% VO_2_peak or 4 minutes of LV-HIT at ∼170% VO_2_peak induce comparable changes in the intra-myocellular environment (glycogen content and signaling activation); correspondingly, training-induced adaptations resulting for these protocols, and other HIT and END protocols are strikingly similar.

## Introduction

Endurance (END) training and interval training, consisting of either sprint interval training (SIT; repeated bouts of ‘all out’ exercise [e.g. Repeated Wingates]) or high-intensity interval training (HIT; repeated bouts of exercise below an all out intensity) result in comparable adaptations in both performance and skeletal muscle metabolism. These include: improvements in maximal oxygen uptake (VO_2_peak) [1]–[7] and aerobic exercise performance [7]–[9], increases in markers of mitochondrial content, oxidative capacity [1]–[3], [8], [10], intramuscular glycogen [8] and triglyceride stores [3], capillary density [4], [6], [11], lipid oxidation [1], [3], [10], and reductions in substrate level (phosphocreatine and glycogen) phosphorylation [1]. Given the markedly reduced training dose (exercise energy expenditure and training time) associated with many high-intensity protocols, these data demonstrate the ability of high intensity exercise to induce physiological adaptations without the requirement for large training volumes.

The potency of HIT high-intensity exercise has recently been demonstrated by several studies employing a low volume- (LV) high-intensity interval training (HIT) protocol requiring a fraction of the training dose (total training time of ∼4 min/day) necessary for END and many other HIT protocols. Similar to other larger dose HIT protocols, studies utilizing LV-HIT have demonstrated improvements in VO_2_peak [9], [12], [13], anaerobic exercise capacity [12], [13], and increased mitochondrial protein content (cytochrome *c* oxidase (COX) subunit I and COX IV)[13] that are equivalent to those induced by END [9], [12]. While there is an abundance of literature demonstrating the efficacy of HIT at inducing adaptations, the mechanisms by which substantially lower doses of high intensity exercise induce adaptations typically associated with high dose END are not completely understood, specifically, similarities/differences in fibre-type specific adaptations induced by HIT and END have not been completely described.

For example, several recent reports suggest that improvements in aerobic exercise capacity and performance following HIT result primarily from peripheral adaptations [14], [15]. These adaptations, specifically the induction of mitochondrial biogenesis, result from the activation of signaling cascades which increase transcription of mitochondrial genes via the activation [16], [17] and/or increased expression of peroxisome proliferator-activated receptor gamma co-activator 1 alpha (PGC-1α) [18]. Given that END and HIT result in similar increases in whole-muscle markers of mitochondrial content and oxidative capacity [1]–[3], [8], [10], it is not surprising that acute activation of signaling pathways, measured in whole-muscle homogenates, following a single bout of END and HIT are also comparable [19]–[21]. However, it is not clear if these whole-muscle measures reflect similar adaptations across distinct fibre populations within a trained muscle, or whether HIT induces adaptations in different populations of fibres. More specifically, whether the well characterized recruitment of type II fibres associated with higher intensities of exercise, including continuous supramaximal [22]–[24] and intermittent maximal [25] exercise result in different fibre recruitment patterns between LV-HIT and END is unknown. Further, whether variations in acute fibre recruitment result in distinctive fibre adaptations following chronic LV-HIT and END is also not known; however, it is possible that different recruitment patterns and fibre-type specific adaptations may partially explain the potency of high intensity exercise and more specifically, LV-HIT.

To examine these issues we performed 2 distinct experiments. The purpose of experiment 1 was to examine: 1) fibre recruitment patterns, as reflected by reductions in glycogen content of each fibre-type population, and 2) whole-muscle and fibre-type specific signalling pathway activation following an acute bout of LV-HIT and END exercise. The purpose of experiment 2 was to examine: 1) changes in aerobic and anaerobic exercise performance, and 2) fibre-type specific adaptations in oxidative and glycolytic capacities, substrate storage and whole-muscle capillary density resulting from 6 weeks of LV-HIT and END training. We hypothesized that an acute bout of LV-HIT would result in the activation of a greater number of type II muscle fibres than END exercise, and that resulting fibre-specific metabolic adaptations following training would reflect these differences in acute fibre recruitment.

## Materials and Methods

### Ethics Statement

All experimental procedures performed on human participants were approved by the Health Sciences Human Research Ethics Board at Queen’s University and conformed to the Declaration of Helsinki. Verbal and written explanation of the experimental protocol and associated risks was provided to all participants prior to obtaining written informed consent.

### Experimental Design

The current study involved the completion of two distinct experiments. Experiment 1 compared fibre specific and whole muscle responses to acute bouts of either low-volume high-intensity interval training (LV-HIT) or moderate-intensity continuous cycle exercise (END) in a randomized crossover design. Experiment 2 examined the impact of a six-week training intervention (END or LV-HIT 4 days/week), on whole body and skeletal muscle fibre specific markers of aerobic and anaerobic capacity. All physiological testing and training for both experiments was performed on a Monark Ergomedic 874 E stationary ergometer (Vansbro, Sweden). Participants were asked to refrain from alcohol and exercise 24 hours before, and caffeine 12 hours before all experimental days in experiment 1 and pre- and post-training testing days in experiment 2.

### Experiment 1

#### Participants

Six recreationally active males volunteered to participate in the study (participant characteristics are presented in Table 1). No participants were involved in more than 3 hours of aerobic exercise (running, jogging, etc.) per week.

**Table 1:** Experiment 1 participant characteristics (N = 6), mean (SD) unless otherwise indicated.

Age (yrs)	20.7(3.8)
Height (cm)	183.7(9.3)
Body mass (kg)	77.8(2.8)
Absolute VO_2_ (mL/min)	4025(306)
Relative VO_2_ (mL/kg/min)	51.9(5.1)
HRpeak (BPM)	196(7)
WRpeak (W)	264.1(41.6)
Mean power (W) LV-HIT (actual)	481.4(60.1)
Mean power (W) LV-HIT (prescribed)	448.0(70.5)
Mean power (W) endurance (actual)	173.7(29.8)
Mean power (W) endurance (prescribed)	172.0(27.1)

Note: LV-HIT mean power represents the average power output during the 8 intervals not including rest periods. BPM, beats per minute; cm, centimeters; kg, kilograms; min, minute; RER, respiratory exchange ratio; s, seconds; W, watts; yrs, years.

#### Physiological testing

On their first visit to the lab participants completed a VO_2_peak incremental ramp test to exhaustion as described previously [26], and height and weight were recorded. Gas exchange was measured throughout the test with a metabolic cart (Moxus AEI Technologies, Pittsburgh, PA), with VO_2_peak and heart rate (HR) peak calculated as the highest value of continuous 30 second averages for each measure during the protocol. Peak O_2_ pulse was calculated by dividing absolute VO_2_peak by HRpeak from the incremental ramp protocol. Work rate (WR) was collected continuously throughout the test and peak aerobic power (WRpeak) was calculated using the average WR from the last 30 seconds of the test.

#### Exercise interventions

Participants reported to the lab in the morning following an overnight fast (≥12 h) after consuming a standardized dinner the night before [Stouffer’s Sauté Sensations Country Beef Pot Roast (540 kcal; 56 g carbohydrate (CHO), 20 g fat, 14 g protein) and 500 mL of 2% milk (260 kcal; 12 g CHO, 5 g fat, 9 g protein)]. A fasted, resting muscle biopsy (Rest) was taken from the vastus lateralis under superficial local anaesthesia (2% lidocaine, with epinephrine) using the Bergstrom needle biopsy technique [27] adapted with suction. An additional incision was made, and covered with sterile gauze, approximately 1 cm from the resting biopsy site to allow for immediate removal of the post-exercise muscle sample. Participants then rested in the lab for 30 minutes or 55 minutes before completing a bout of END or LV-HIT, respectively. Immediately following each exercise bout a second muscle biopsy was taken (Ex). The LV-HIT exercise protocol was performed as described previously [12], [13]. Briefly, participants completed eight 20-second intervals at 170% of VO_2_peak separated by 10 seconds of rest eight times, for a total of four-minutes. During rest periods participants cycled against no load at a cadence of their choice. The END exercise protocol consisted of 30 minutes of continuous cycling at 65% of VO_2_peak. Each exercise bout was performed at the same time of day for all subjects and were performed one week apart (7 days).

### Experiment 2

#### Participants

Nineteen recreationally active males (n = 16) and females (n = 3) volunteered to participate in this study (participant characteristics are presented in Table 1). No participants were involved in more than 3 hours of aerobic exercise (running, jogging, etc.) per week or involved in any structured training program within the past six months. Participants were VO_2_peak-matched and assigned to either the LV-HIT (n = 10) or endurance (n = 9) group.

#### Training interventions

All participants completed training four days a week for six weeks (week 4 only had 3 training sessions due to the mid-training VO_2_peak test for a total of 23 sessions). Both the LV-HIT and END training protocols were the same as those utilized in experiment 1 (see above for descriptions). Participants descended and ascended 4 flights of stairs as a warm-up prior to all training sessions. Revolutions per minute (RPM) data was collected for each training session in order to confirm training intensity.

#### Physiological testing

During baseline testing (Pre) participants reported to the lab in the morning following an overnight fast (≥8 h). Participants were fed a standardized breakfast [plain bagel (190 kcal; 1 g fat, 36 g CHO, 7 g protein) with 15 g of peanut butter (90 kcal; 8 g fat, 4 g CHO, 3 g protein) and 200 mL of apple juice (90 kcal; 0 g fat, 22 g CHO, 0 g protein)] and rested for 1 hour before a muscle biopsy was taken from their vastus lateralis under superficial local anaesthesia (2% lidocaine, with epinephrine) using the Bergstrom needle biopsy technique (1) adapted with suction.

Forty-eight hours following the muscle biopsy participants returned to the lab to complete a VO_2_peak incremental ramp test to exhaustion as described previously [26]. Following completion of the VO_2_peak test, participants rested for 30 minutes before completing a repeated Wingate protocol consisting of 2 minutes of load-less cycling followed by four 30 second Wingates (7.5% body weight), separated by 4.5 minutes of load-less cycling. RPM was collected continuously and peak (first 5 second average) and mean power (average for total 30 seconds) were determined for the first Wingate. Total work performed during all 4 Wingate bouts was also calculated. Twenty-four hours after the VO_2_peak and Wingate tests, participants reported to the lab and completed a 500 kcal time to completion (TTC) trial at a self-selected cadence as quickly as possible as described previously [28], [29].

A VO_2_peak incremental ramp test to exhaustion was performed half way through training (Mid) on the first day of week 4 in order to adjust training loads. As a result participants only completed 3 training sessions in week 4. Post-training (Post) testing began 72 hours following the last training session of week 6 and was conducted in an identical manner as the baseline testing. (See above for details).

### Immunofluorescent and Histochemical Analysis

Immunofluorescent analysis of myosin heavy chain isoforms was performed as previously described [30] using primary antibodies against myosin heavy chain (MHC) I (BA-F8), MHCIIa (SC-71), and MHCIIx (6H1) (Developmental Studies Hybridoma Bank, Iowa City, IA, USA), followed by isotype-specific fluorescent secondary antibodies. This allowed for the identification of type I (blue), type IIA (green), type IIX (red), as well as hybrid fibre types (IIAX). For all analyses type IIX and type IIAX fibres were analyzed together (type IIAX/IIX) given their relatively low percentage. Fibre type composition was determined by counting all fibres within a muscle cross-section.

Quantification of capillary density [31] and fibre-type specific phosphorylated-AMP-activated protein kinase alpha (p-AMPKα) [32] was adapted from previous work. Briefly, sections were fixed in 4% paraformaldehyde for 10 minutes, followed by permeabilization with 0.5% TritonX-100 for 30 minutes, and then blocked in 10% goat serum for 30 minutes. Sections were incubated overnight in 5% goat serum with the appropriate primary antibodies specific for the endothelium (PECAM) and sarcolemma (dystrophin) (Developmental Studies Hybridoma Bank, Iowa City, IA, USA) or p-AMPKα (Thr^172^) (Cell Signaling Technology, Danvers, MA, USA). After 3×5 minute washes in PBS, sections were incubated for 1 hour in 5% goat serum with the appropriate fluorescent secondary antibodies (Life Technologies, Burlington, ON, CA).

For all immunofluorescent procedures, sections were mounted with Prolong Gold Antifade Reagent (Life Technologies, Burlington, ON, CA) and imaged the following day. All sections were visualized with an Axio Observer Z1 microscope (Carl Zeiss, Jena, TH, DE). Individual images were taken across the entire muscle cross-section and assembled into a composite panoramic image using AxioVision software (Carl Zeiss). Compiled images were matched to fibre-type images and ∼30 of each fibre type were randomly selected and analyzed. Data were expressed relative to the values obtained in type I fibers, which were assigned a reference value of 1.0, and reported as mean optical density in arbitrary units (AU). This method was utilized for all immunofluorescent and histochemical analyses except for the determination of capillary density where 3 separate areas per sample were analyzed for number of capillaries. Fibre-specific p-AMPKα fluorescence was performed in ImageJ (National Institutes of Health, Bethesda, MD, USA) and was determined by subtracting the average fluorescence of the slide background (>5 per slide) from each individual fibre’s fluorescence.

As a general indicator of oxidative and glycolytic potential, histochemical staining for succinate dehydrogenase (SDH) [33] and α-glycerophosphate dehydrogenase (GPD) [34] activity was performed [30]. Intramusclar triglyceride (IMTG) content was determined with Oil Red O (ORO) staining [35], whereas glycogen content was determined using the Periodic Acid Schiff (PAS) [36]. Images were acquired with a bright field Nikon microscope linked to a PixelLink digital camera. Individual images were taken across the entire muscle cross-section and assembled into a composite panoramic image using Microsoft Image Composite Editor (ICE) (Microsoft, Redmond, WA, USA). Image analysis was performed in ImageJ by converting color images to 8-bit, and calculated by subtracting background staining from the mean grey value of individual fibres. Estimated oxidative and glycolytic capacities were calculated by summing the calculated capacities of each fibre type together. Capacities for each fibre type were calculated by multiplying the mean SDH (oxidative) or GPD (glycolytic) activity for each fibre-type by the percent of that fibre present within each participant’s sample.

### Western Blotting

Samples were homogenized, total protein was determined, and sample loading, separation and transfer were performed as described previously [29]. All phosphorylated protein contents are expressed relative to total protein content at rest. Samples were run in duplicate and equal protein loading was confirmed with GAPDH analysis. For protein detection, commercially available antibodies were used for GAPDH (Millipore, Billerica, MA, USA), p-p38 MAPK (Thr^180^/Tyr^182^), p-38 MAPK, p-AMPKα (Thr^172^), AMPKα, p-ACC (Ser^79^), and ACC (Cell Signaling Technologies, Danvers, MA, USA). Membranes were blocked with 5% bovine serum albumin (BSA) in Tris-buffered saline with Tween-20 (TBS-T) (0.1%) and were immunoblotted with primary antibodies (1∶1000). Proteins were visualized by chemiluminescence detection, according to the manufacturer’s instructions (Millipore, Billerica, MA, USA). Blots were quantified using the Fluorochem HD2 System (Cell Biosciences, Santa Clara, CA, USA).

### RNA Extraction

RNA was extracted using the TRIzol/RNeasy method (Qiagen Sciences, Valencia, CA, USA). Briefly, approximately 20 mg of frozen muscle was added to Lysing Matrix D tubes (MP Biomedicals, Solon, OH, USA), containing 1 mL of TRIzol Reagent (Life Technologies, Burlington, ON, Canada). Muscle samples were homogenized using the FastPrep-24 Tissue and Cell Homogenizer (MP Biomedicals, Solon, OH, USA) for 40 sec at a setting of 6 m/sec. 200 µl of chloroform reagent was added to homogenized samples (Sigma-Aldrich, Oakville, ON, CA), mixed for 15 sec, incubated at room temperature for 5 minutes and centrifuged for 10 minutes at 12000 g at 4°C. The aqueous phase was transferred to a new tube and equal amounts of 70% ethanol was added and mixed. The mixture was transferred into an RNeasy mini-spin column and RNA was purified following the commercially available E.Z.N.A. Total RNA Kit 1 (Omega Bio-Tek, Norcross, GA, USA) as per manufacturer’s instructions. The RNA concentration and purity was quantified using the Nano-Drop 1000 spectrophotometer (Thermo Fisher Scientific, Rockville, MD, USA). RNA integrity was assessed using a bioanalyzer (Agilent 2100 Bioanalyzer; Agilent Technologies). Average RNA Integrity Number (RIN) values were 6.4.

### Quantitative Real-time PCR

Individual samples were reverse transcribed using the commercially available high capacity cDNA reverse transcription kit (Applied Biosystems, Foster City, CA, USA), according to the manufacturer’s instructions, using an Eppendorf Mastercycler epgradient thermal cycler (Eppendorf, Mississauga, ON, CA). Reactions were run using RT^2^ real-time SYBR Green qPCR master mix (SABioscience; Qiagen Sciences, Germantown, MD, USA), prepared using the epMotion 5075 Eppendorf automated pipetting system (Eppendorf, Mississauga, ON, CA) and carried out in duplicate with an Eppendorf realplex^2^ Master Cycler epgradientS (Eppendorf, Mississauga, ON, CA). Primer sequences are shown in Table 2. Relative mRNA expression was calculated using the ΔCt method and fold changes from PRE was calculated using the ΔΔCt method [37]. Gene expression was normalized to the housekeeping gene GAPDH.

**Table 2:** Quantitative RT-PCR primer sequences.

	Forward Primer	Reverse Primer
PGC-1α	Cacttacaagccaaaccaacaact	caatagtcttgttctcaaatgggga
SIRT1	Agaacatagacacgctggaaca	caagatgctgttgcaaaggaacc
GCN5	Aaggaccccgaccagctcta	gggaagcggatgacctcgta
RIP140	Gttccactcagcccagcagt	agaccctgcacagcccaagt
GAPDH	cctcctgcaccaccaactgctt	gaggggccatccacagtcttct

Note: GAPDH, glyceraldehyde 3-phosphate dehydrogenase; GCN5, general control non-derepressible 5; PGC-1α, peroxisome proliferator-activated receptor gamma coactivator 1 alpha; RIP140, receptor-interacting protein 140; SIRT1, silent mating type information regulation 2 homolog 1.

### Statistical Analysis

For both experiments, a two-way, repeated measures ANOVA was used to compare the effect of condition (END and LV-HIT) and time (Rest/Ex and Pre/Post) for all muscle derived data and pre- to post-training physiological testing data from experiment 2. All fibre-type specific analyses were performed within a particular fibre-type. Differences in fibre-type specific expression of p-AMPKα were determined using paired students t-tests comparing mean fluorescence between fibre-types at that time point (Rest and Ex). For estimated glycolytic capacity, where no interaction effect or main effects of training were observed, paired student t-tests were utilized to examine within group changes. All statistical analysis was performed using GraphPad Prism v 5.01 (GraphPad Software Inc., La Jolla, CA, USA). Statistical significance was accepted at p<0.05 and all data are presented as means ± SD.

## Results

### Experiment 1

#### Exercise bout characteristics

All participants completed each exercise protocol at a work rate corresponding to 65.7±3.8% and 183.2±9.5% VO_2_peak for the END and LV-HIT exercise bouts, respectively. Mean prescribed and actual WR for both protocols are presented in Table 1.

#### Immunofluorescent and histochemical analysis

**Figure 1:**
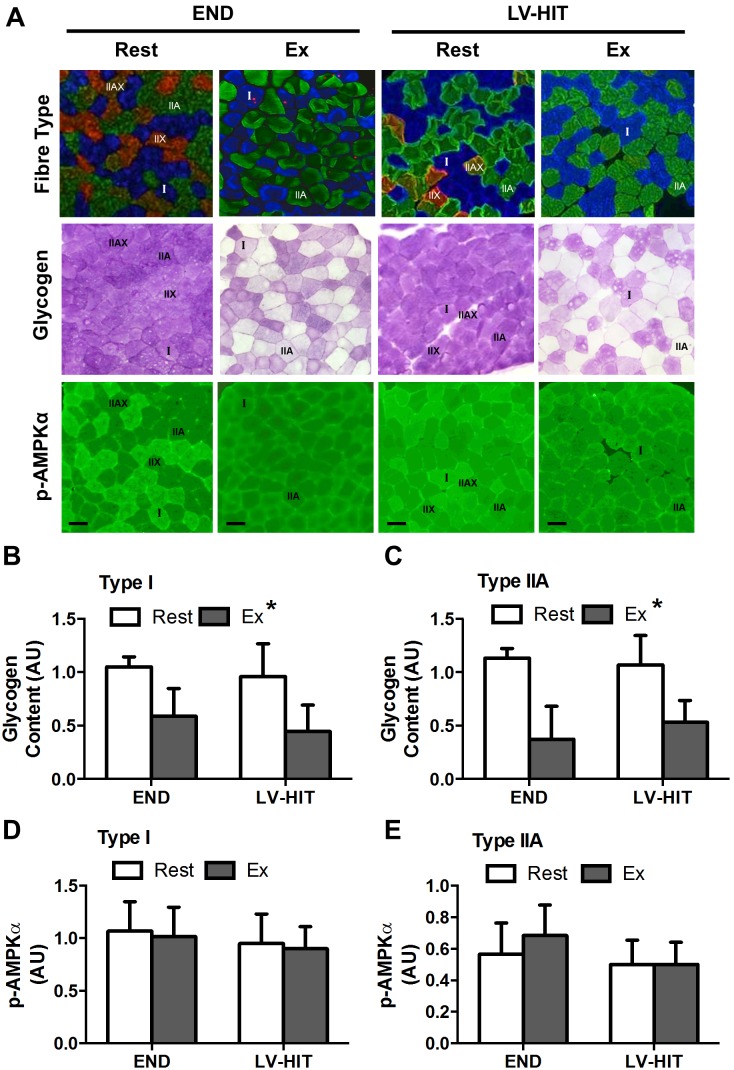
Effects of acute exercise on fibre-type specific glycogen depletion and phosphorylation of AMPKα. A: Representative slides of immunofluorescent fibre-type analysis (blue fibres are type I, green are type IIA, red/green are type IIAX/IIX) with serial sections of glycogen (periodic acid-Schiff staining) and p-AMPKα Thr^172^ content before (Rest) and immediately after (Ex) an acute bout of END or LV-HIT. B–C: Fold change in glycogen content from Rest and Ex in type I and IIA fibres. D–E: Fold change in p-AMPKα content from Rest and Ex in type I and IIA fibres. Bars represent 100 µm. AU, arbitrary units; µm, micrometre. *Significant (p<0.05) main effect of exercise.

Representative images are presented in Figure 1A. While no interaction effect was observed for either type I or type IIA fibres, a significant (p<0.05) main effect of exercise for muscle glycogen content was present for type I (END–42%, LV-HIT–55%; Fig. 1B) and IIA (END–72%, LV-HIT–49%; Fig. 1C) fibres. As a result of type IIAX/IIX (END, n = 3; LV-HIT, n = 4) fibres not being present in all samples, they were excluded from analysis.

There was no interaction effect, or effect of exercise for p-AMPKα content in type I (Fig. 1D) or type IIA (Fig. 1E) fibres (see representative slides Fig. 1A). A similar lack of effect of exercise on p-AMPKα content was observed with whole muscle Western Blot analysis (see below). Type I fibres expressed significantly (p<0.05) greater levels of p-AMPKα than both type IIA and IIAX/IIX fibres at rest and post-exercise.

#### Western blotting

**Figure 2:**
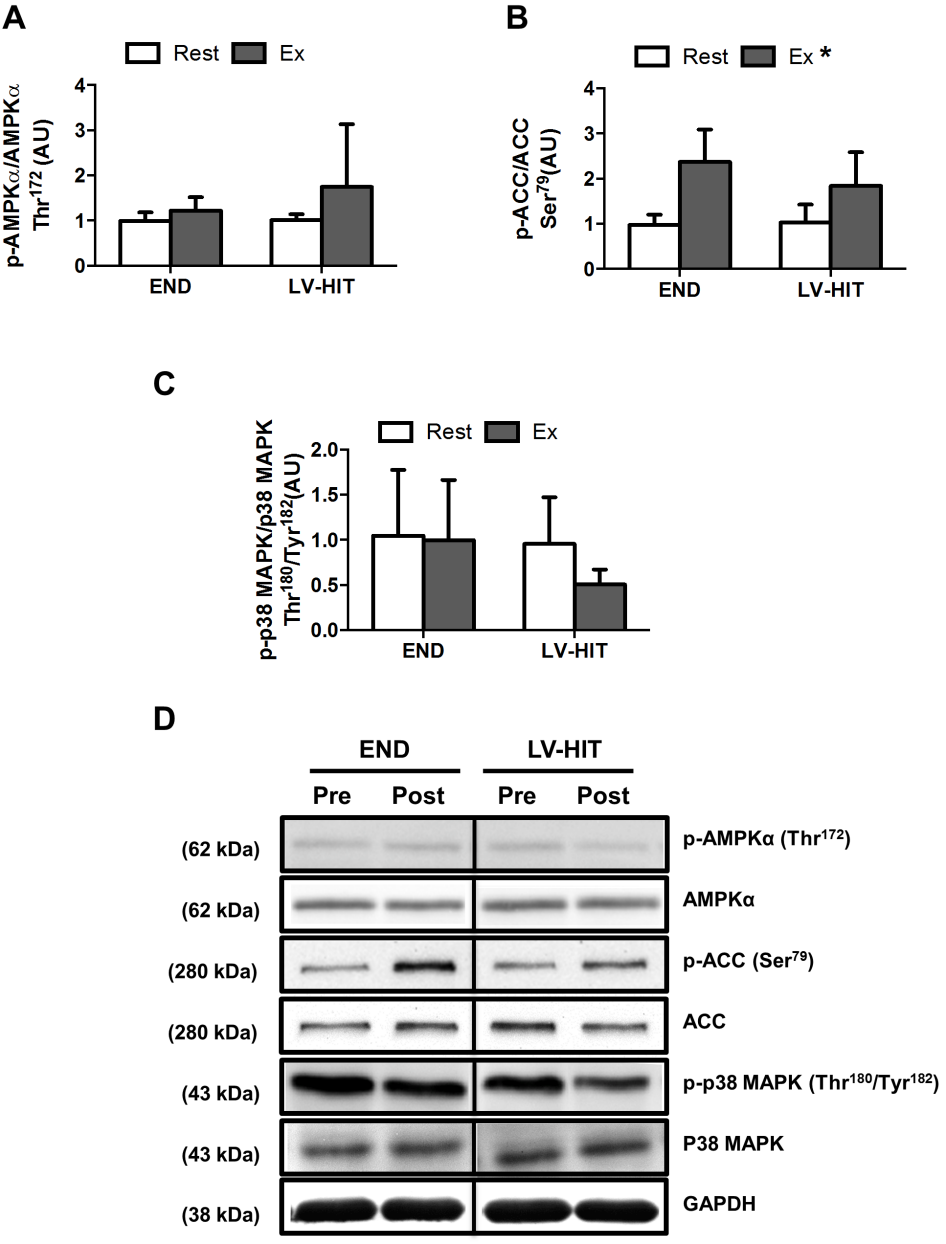
Effects of an acute bout of endurance (END) and low-volume high-intensity interval training (LV-HIT) on whole-muscle signaling. A–D: Fold change in p-AMPKα Thr^172^ (A), p-ACC Ser^79^ (B) and p38 MAPK Thr^180^/Tyr^182^ (C) content from Rest and Ex. D: Representative western blots, including loading control are also shown. AU, arbitrary units. *Significant (p<0.05) main effect of exercise.

There was no effect (Fig. 2A and C) of exercise on p-AMPKα Thr^172^ or p-p38MAPK Thr^180^/Tyr^182^ (see representative blots, Fig. 2D). However a main effect (p<0.05) of exercise was observed for p-ACC Ser^79^ (Fig. 2B).

### Experiment 2

#### Aerobic exercise performance and oxidative capacity

**Figure 3:**
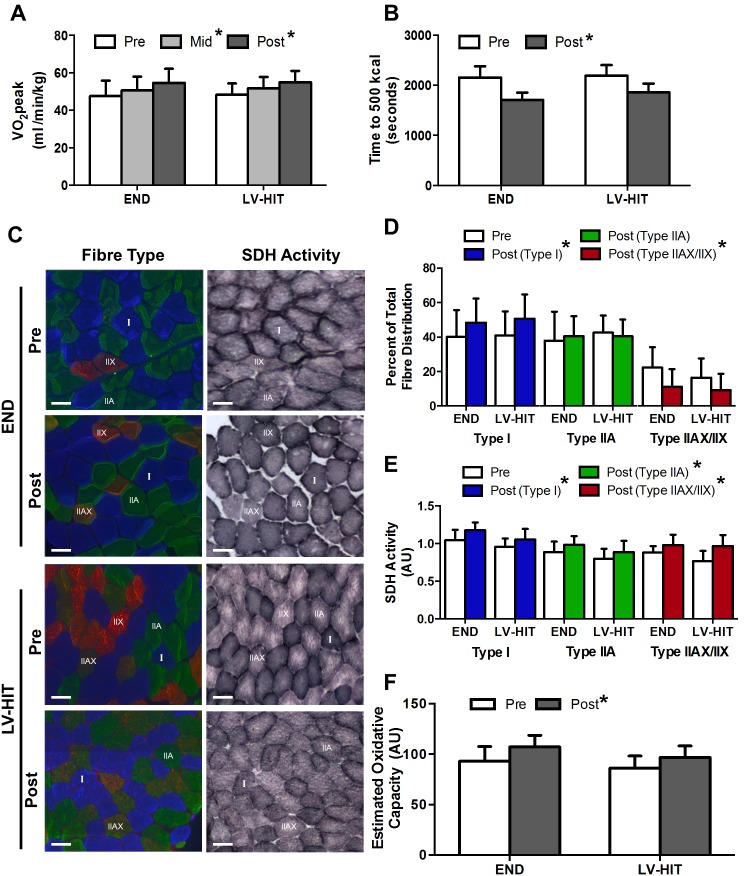
Effects of training (LV-HIT) on aerobic exercise performance, fibre-type distribution and fibre specific oxidative capacity. Relative VO_2_peak (A) and time to 500 kcal (B) at Pre, Mid, and Post for both END and LV-HIT groups are shown. C: Representative slides of immunofluorescent fibre-type analysis (blue fibres are type I, green are type IIA, red/green are type IIAX/IIX) with serial sections of succinate dehydrogenase (SDH) activity Pre and Post END or LV-HIT. D: Percentage of total fibre distribution of type I, IIA and IIAX/IIX fibres Pre and Post END or LV-HIT. E: Fibre-type specific fold change in SDH activity post-training compared to pre-training in type I, IIA and IIAX/IIX fibres. F: Absolute change in estimated oxidative capacity. Bars represent 100 µm. AU, arbitrary units; µm, micrometre. *Significant (p<0.05) main effect of exercise.

A main effect of training (p<0.05) on relative VO_2_peak (Fig. 3A) was observed at both Mid and Post testing (Percent change from pre: END: +15.5%, LV-HIT: +13.9%). A significant (p<0.05) main effect of training on absolute VO_2_peak and peak HR was also demonstrated, while no effect of training was observed for WRpeak (Table 3). A significant (p<0.05) main effect of training was also observed for the time to complete the 500 kcal test (Percent change from pre: END: +20.4%, LV-HIT: +15.3%; Fig. 3B).

**Table 3:** Experiment 2 participant characteristics (N = 19) for low volume high-intensity interval (LV-HIT) training (n = 10) and endurance (END) training (n = 9) at baseline (pre), mid-training (mid) and following 6 weeks of training (post); mean (SD) unless otherwise indicated.

	END			LV- HIT		
	Pre	Mid	Post	Pre	Mid	Post
Age (yrs)	21(4)	-	-	21(2)	-	-
Height (cm)	180(7)	-	-	175(10)	-	-
Body mass (kg)	74(9)	74(8)	74(9)	71(17)	71(17)	71(17)
Absolute VO_2_ (mL/min)*	3534(768)	3743(715)	4050(760)	3440(913)	3703(1007)	3910(1094)
Relative VO_2_ (mL/kg/min)*	47.6(8.3)	50.6(7.4)	54.6(7.6)	48.3(6.1)	51.8(6.0)	54.9(6.2)
HRpeak (bpm)*	194(11)	190(9)	191(9)	195(7)	192(6)	193(3)
WRpeak (W)	255(62)	285(56)	281(58)	250(58)	262(53)	258(63)
Wingate peak power (W)*^†^	718(142)	-	704(147)	654(146)	-	718(184)
Wingate mean power (W)*^†^	570(119)	-	594(122)	498(141)	-	580(131)

Note: *Main effect of training, p<0.05; ^†^significant interaction, p<0.05. BPM, beats per minute; cm, centimeters; kg, kilograms; min, minute; mL, milliliter; RER, respiratory exchange ratio; s, seconds; W, watts; yrs, years.

A main effect of training (p<0.05) was demonstrated for fibre distribution of type I and type IIAX/IIX fibres, with the percentage of type I fibres increasing and type IIAX/IIX fibres decreasing following training (Fig. 3D). No effect of training was demonstrated for the percentage fibre distribution for type IIA (Fig. 3D) fibres (see representative slides Fig. 3C). A main effect of training (p<0.05) was observed for fold change in SDH activity in type I (n = 9), type IIA (n = 9) and type IIAX/IIX (n = 6)(Fig. 3E, see representative slides Fig. 3C). A main effect of training (p<0.05) was also observed for estimated oxidative capacity (Fig. 3F).

#### Cardiovascular capacity and capillary density

**Figure 4:**
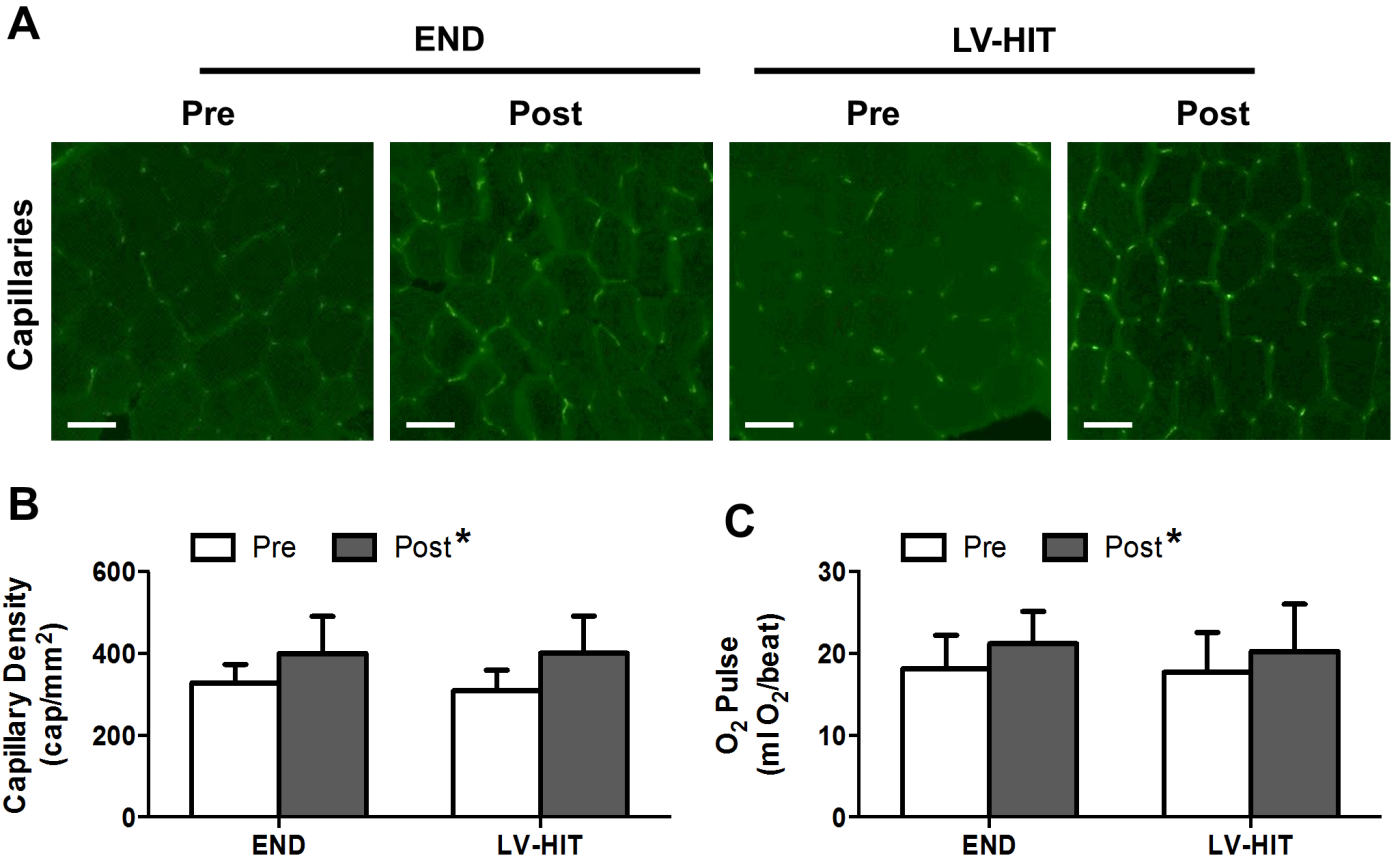
Effects of 6 weeks of endurance (END) and low-volume high-intensity interval training (LV-HIT) on cardiovascular capacity and capillary density. A: Representative slides of capillarisation Pre and Post training. B: Absolute change in capillary density (capillaries/mm^2^). C: Change in O_2_ pulse (mL O_2_/beat). Bars represent 100 µm. mL, milliliter; mm, millimeter; O_2_, oxygen; µm, micrometre. *Significant (p<0.05) main effect of exercise.

A main effect of training (p<0.05) was observed for capillary density (Fig. 4B) (see representative slides Fig. 4A) and O_2_ pulse (Fig. 4C).

#### Anaerobic exercise performance and glycolytic capacity

**Figure 5:**
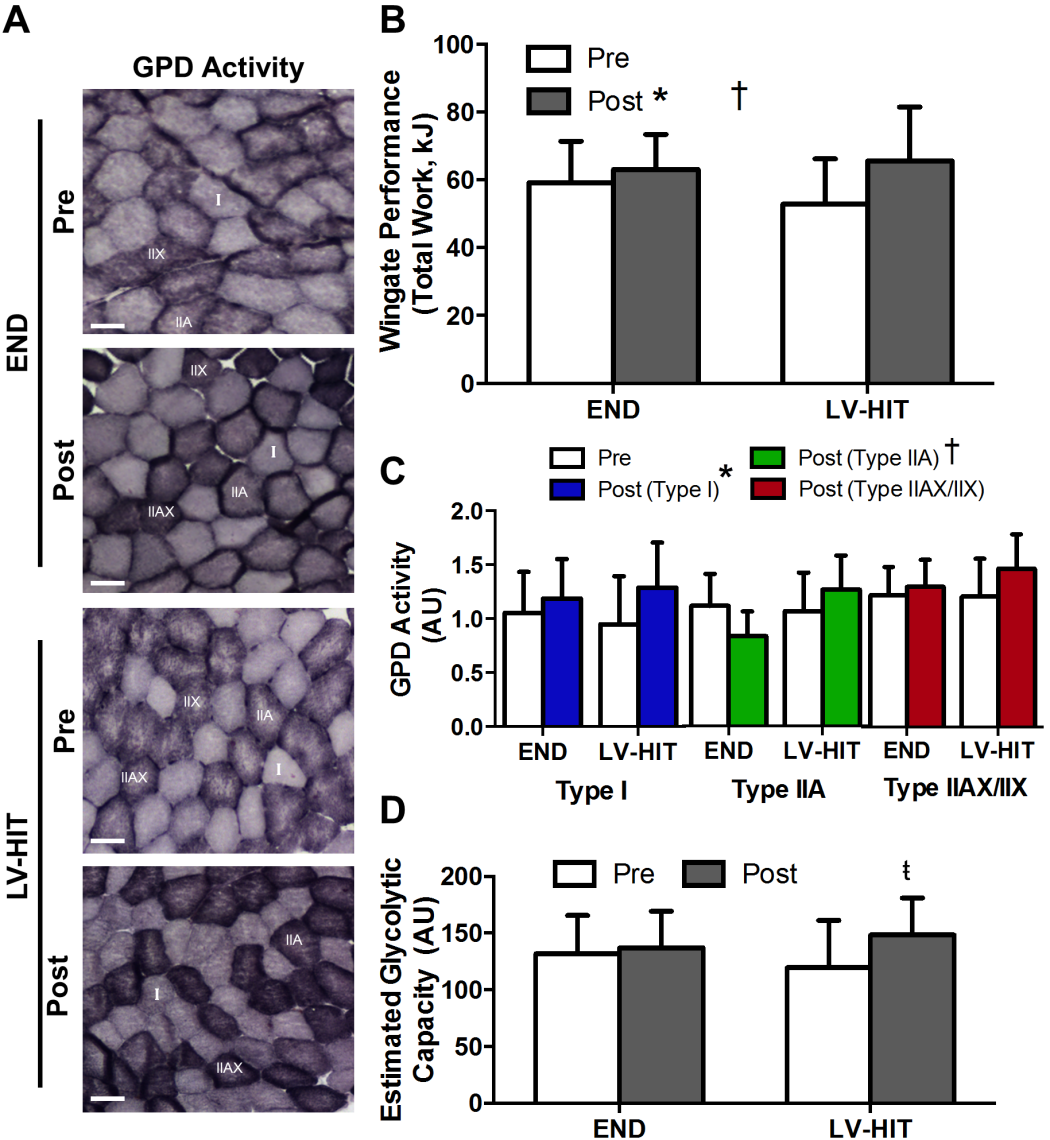
Effects of endurance (END) and low-volume high-intensity interval training (LV-HIT) on anaerobic exercise performance and glycolytic capacity. A: Representative slides of α-glycerophosphate dehydrogenase (GPD) activity Pre and Post training (see Fig. 3C for corresponding fibre-type slides). B: Total work performed during all 4 Wingate bouts. C: Fibre-type specific fold change in GPD activity post-training compared to pre-training in type I, IIA and IIAX/IIX fibres. D: Absolute change in estimated glycolytic capacity. Bars represent 100 µm. AU, arbitrary units; µm, micrometre. †Significant (p<0.05) group by time interaction, *Significant (p<0.05) main effect of exercise, 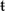
Significant (p<0.05) within group difference (derived from paired t-test).

A significant interaction effect (p<0.05) and a main effect of training (p<0.05) were demonstrated for both peak and mean power in the first Wingate completed (Table 3), and for total work completed across all 4 Wingates (Fig. 5B). A main effect of training (p<0.05) was observed for fold change in GPD activity in type I fibres (see representative slides Fig. 5A). There was no effect of training observed for GPD activity in type IIA and IIAX/IIX fibres, while a significant (p<0.05) interaction was demonstrated for type IIA fibres (Fig. 5C). There was no main effect of training for estimated glycolytic capacity (Fig. 5D); however, paired students t-tests demonstrated a significant (p<0.05) increase in estimated glycolytic capacity post-training (Post) in the LV-HIT group only.

#### Intramuscular glycogen and triglyceride content

**Figure 6:**
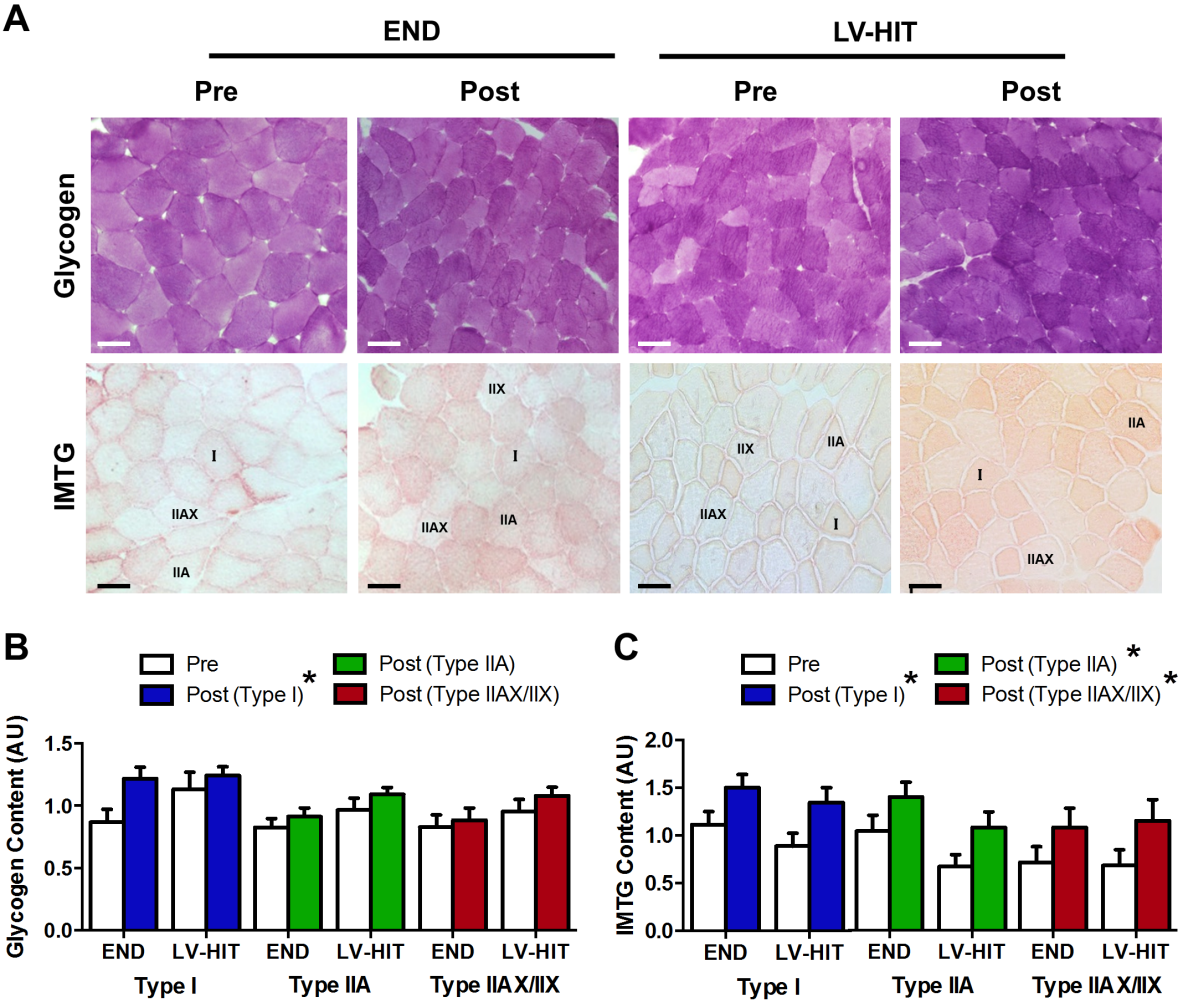
Effects of endurance (END) and low-volume high-intensity interval training (LV-HIT) on intramuscular glycogen and triglyceride storage. A: Representative slides of glycogen (periodic acid-Schiff reaction) and intramuscular triglyceride (IMTG) content (Oil red O staining) Pre and Post training (see Fig. 3C for corresponding fibre-type slides for IMTG stain only). B. Fibre-type specific fold changes in glycogen content in type I, IIA and IIAX/IIX fibres. C. Fibre-type specific fold changes in IMTG content in type I, IIA and IIAX/IIX fibres. Bars represent 100 µm. AU, arbitrary units; µm, micrometre. *Significant (p<0.05) main effect of exercise.

A main effect of training (p<0.05) was observed for glycogen content in type I fibres. There was no main effect of training for percent change of glycogen content in type IIA and IIAX/IIX fibres (Fig. 6B, see representative slides Fig. 6A). A main effect of training (p<0.05) was observed for IMTG content in type I, IIA and IIAX/IIX fibres (Fig. 6C, see representative slides Fig. 6A).

#### mRNA expression

Unpaired students t-tests demonstrated no difference in fold change of PGC-1α (END [n = 9]: 1.46±1.10 AU; HIT: 0.87±0.40 AU), general control non-derepressible (GCN) 5 (END [n = 9]: 1.04±0.74 AU; HIT: 0.81±0.45 AU) and receptor-interacting protein (RIP) 140 mRNA (END [n = 9]: 0.98±0.42 AU; HIT: 0.81±0.37 AU) expression between groups, and a significantly (p<0.05) higher fold change of silent mating type information regulation 2 homolog 1 (SIRT1) mRNA expression in the END group only (END [n = 9]: 1.19±0.57 AU; HIT: 0.74±0.23 AU) following training.

## Discussion

The present study examined both fibre recruitment patterns in response to acute bouts of LV-HIT and END, and fibre-type specific adaptations following 6 weeks of either LV-HIT or END training. In experiment 1, we observed comparable changes in whole-muscle cellular signaling and glycogen depletion derived estimates of fibre recruitment following LV-HIT and END. Consistent with similar responses following the acute exercise bouts, results from experiment 2 demonstrate that 6 weeks of LV-HIT and END training induced comparable increases in aerobic capacity (VO_2_peak) and exercise performance (TTC trial), changes in fibre-type distribution, fibre-type specific oxidative and glycolytic capacity, glycogen and IMTG storage, and whole-muscle capillary density. Interestingly, LV-HIT induced greater improvements in anaerobic exercise performance and estimated whole-muscle glycolytic capacity than END.

### Experiment 1: Fibre Recruitment Patterns during LV-HIT and END

Whole-muscle measures of signaling pathway activation following acute exercise [19]–[21], and chronic increases in mitochondrial protein/oxidative capacity [1]–[3], [8], [10] are typically comparable following interval training and END. The current study was designed to investigate whether these similarities in whole-muscle measures are masking differences in fibre-type specific adaptations that may help explain the potency of high-intensity exercise and, more specifically, LV-HIT. Fibre recruitment, as indicated by fibre-type specific glycogen depletion, following steady state submaximal exercise is dependent on exercise intensity with type I fibres being preferentially recruited during low intensity exercise and type II fibres being progressively recruited with increasing exercise intensity [24], [38], [39]. Based on this relationship, we hypothesized that LV-HIT (∼170% VO_2_peak WR) would result in a greater number of recruited fibres (both type I and II) than END (∼65% VO_2_peak WR) which would recruit primarily type I fibres.

Contrary to our hypothesis, we observed equivalent reductions (p<0.05) in glycogen content in type I and IIA fibres following both protocols (Fig. 1B and C), suggesting that fibre recruitment during LV-HIT and END is comparable. While these data are consistent with both type I and IIA fibres being recruited during supramaximal exercise [22]–[25], the observed glycogen depletion in both fibre types following END is somewhat surprising [24], [25], [38]. Regardless, in combination with previous demonstrations of type IIA fibre glycogen depletion during submaximal exercise [38], [39] our results suggest that there is a threshold intensity associated with the recruitment of a significant percentage of type IIA fibres and that this threshold is below ∼65% of VO_2_peak. Thus, it would appear that similarities in whole-muscle measures of signaling pathway activation between interval training and END observed previously [19]–[21], and between LV-HIT and END observed in the current study (Fig. 2), likely result from similar fibre recruitment patterns.

Given the equivalent fibre recruitment between LV-HIT and END, and the substantially greater power generation during LV-HIT, it seems reasonable to assume that a greater relative intensity of signal activation would be experienced within individual fibres during LV-HIT. It is therefore somewhat surprising that fibre-type specific phosphorylation of AMPK (Fig. 1D and E), whole-muscle phosphorylation of ACC (a downstream target of AMPK; Fig. 2) and p38 MAPK were not different between conditions. This finding is consistent with a previous report demonstrating similar activation of intramuscular signaling between END (∼70% VO_2_max) and submaximal (∼90% VO_2_max) HIT [19], but not with the recent observation that signaling events are intensity dependent when continuous submaximal exercise (∼40 vs. ∼80% VO_2_peak) was compared [40]. Further, the finding that AMPK and p38 phosphorylation were not increased following LV-HIT is in contrast to observation made following SIT [18]. The reasons for this discrepancy in intramuscular signalling are unclear, however the brevity of the LV-HIT protocol utilized in the current study, compared to the longer duration of SIT protocols utilized previously, suggests that the duration of exercise may be an important factor underlying the activation of these pathways. Collectively, these results suggest that END exercise at an intensity >65% VO_2_peak results in both similar fibre recruitment and activation of intramuscular signaling pathways as more intense, lower dose HIT. Additionally, it appears that the intensity/duration threshold required to activate intracellular signaling events associated with the induction of mitochondrial biogenesis within an individual muscle fibre is achieved by both LV-HIT and END exercise at intensities equal to, or greater than 65% VO_2_peak and a duration of at least 30 minutes [19], [40]. This assertion is supported by the presence of similar levels of PGC-1α mRNA and other genes related to mitochondrial biogenesis following 90 minutes of continuous submaximal (∼65% VO_2_peak) and supramaximal (∼120% VO_2_peak) interval exercise [20], [21] in both type I and IIA fibres [21].

In summary, experiment 1 of the current study demonstrated that recruitment of muscle fibres (type I and IIA) during a single bout of LV-HIT or END are similar, as is activation of intracellular signaling pathways. Given that type I and IIA fibres represent ∼90% of the total fibre population of the vastus lateralis (Fig. 3D) [30], the assumption that changes in both type I and type IIA fibres are accurately reflected in whole-muscle measures of intracellular signaling appears to be appropriate.

### Experiment 2: Fibre Specific Adaptations to LV-HIT and END Training

Exercise training increases the maximal activity and content of mitochondrial enzymes involved in oxidative metabolism, capillary density, and intramuscular triglyceride and glycogen stores [1]–[4], [6], [8], [10], [11]. Collectively, these adaptations contribute to improved skeletal muscle oxidative potential, fatty acid oxidative capacity, and improve aerobic capacity and exercise performance (VO_2_peak, time to fatigue trials, etc.). Based on the recent argument that improvements in aerobic capacity and performance following interval training are driven primarily by peripheral adaptations [14], [15], and the previously discussed hypothesis that LV-HIT would recruit additional different muscle fibres than END, we examined fibre-type specific adaptations to both LV-HIT and END training.

### Aerobic Exercise Performance and Oxidative Capacity

As has been previously observed [9], [12], we demonstrated similar improvements in VO_2_peak and aerobic exercise performance (Fig. 3A and B) following 6 weeks of LV-HIT and END. Consistent with these whole body adaptations, comparable increases in SDH activity, a mitochondrial protein correlated with mitochondrial content [41], were observed across all fibre-types following both LV-HIT and END (Fig. 3E). Additionally, changes in whole muscle gene expression of proteins associated with mitochondrial biogenesis were similar following both training protocols. While others have reported comparable increases in fibre specific cytochorome c oxidase (COX) protein content following SIT and END [3], COX is not highly correlated with mitochondrial content (r = 0.55) [41]. Our results supports these assumptions but are derived from SDH activity, a protein which correlates much more highly with mitochondrial content (r = 0.73) [41]. These adaptations are also consistent with the muscle fibre activation patterns observed during a single bout of LV-HIT and END in experiment 1 suggesting that recruitment of fibres during repeated acute bouts of LV-HIT and END (i.e. training) results in increases in oxidative capacity within those fibres. The results also further strengthen the assertion made above that the intensity/duration threshold required to induce increases in oxidative capacity within a given fibre are reached by both the LV-HIT and END protocols utilized in the present study.

Further, both groups demonstrated an increased proportion of type I fibres concomitant with a reduction in type IIAX/IIX fibres (Fig. 3D). While several studies have demonstrated myofiber transitions resulting in a greater percentage of oxidative fibres (type I) following HIT and END training [42], [43], our results are not consistent with several reports demonstrating increases in type IIA fibres with concurrent reductions in type I fibres following several weeks of SIT [44]–[47]. While the exact mechanism(s) responsible for contraction-induced changes in myofiber composition remain to be completely described, contraction frequency has been demonstrated to influence fibre-type synthesis [48] with high-frequency activation inducing the synthesis of fast twitch myosin. Importantly, our END and LV-HIT protocols required participants to cycle at the same speed (80 RPM), while the load was changed in order to manipulate training intensity. This may explain why we observed increases in type I fibres and reductions in type IIAX/IIX fibres following 6 weeks of both LV-HIT and END, as opposed to those studies employing SIT, a training protocol typically demanding maximal pedaling frequency and contraction rates [44]–[47].

Combined, the increases in fibre-type specific SDH activity and the increase proportion of type I fibres resulted in an increase in estimated whole-muscle oxidative capacity (Fig. 3F) following both training protocols. These results support prior observations of comparable increases in whole-muscle markers of oxidative capacity and enzymes associated with oxidative metabolism [1], [3], [18], [49], [50] following HIT or SIT and END training. We also observed similar increases in capillary density (Fig. 4B), data which are consistent with previous studies reporting comparable increases in capillary density following SIT and END [4], [6], [11]. While these results are in agreement with improvements in aerobic capacity and performance following interval training being mediated by peripheral adaptations, the similar changes in estimated oxidative capacity, capillary density and O_2_ pulse in both groups (Fig. 4C) are not consistent with suggestions that increases in VO_2_peak are supported by different mechanisms following LV-HIT and END [14].

### Anaerobic Exercise Performance and Glycolytic Capacity

Six weeks of LV-HIT and END resulted in improved anaerobic exercise performance (total work; Fig. 5B) and anaerobic capacity (peak and mean power; Table 3). Importantly, improvements for all indices of anaerobic performance/capacity were greater for LV-HIT than END. Consistent with these results, changes in GPD activity were similar in type I fibres (Fig. 5C) while a significant interaction effect was observed in type IIA fibres such that an increased GPD activity was only observed in this fibre population following LV-HIT. Additionally, our estimate of whole-muscle glycolytic capacity demonstrated a trend towards an increase following LV-HIT only (no main effect of training; paired t-test significant within LV-HIT only; Fig. 5D). These results are consistent with previous demonstrations that HIT and SIT improve anaerobic exercise performance [1], [49], while changes in glycolytic enzyme activity have been observed following SIT but not END [5], [50]–[52]. While the exact mechanisms responsible for our observed differences in anaerobic exercise capacity and performance between groups are unknown, it appears that they may be attributable to factors beyond the peripheral skeletal muscle adaptations examined in the present study. One such peripheral factor could be an improved skeletal muscle lactic acid buffering capacity, which has been observed following SIT [8], but not HIT [53]. Another potential mechanism may be differences in adaptations of central origin, such as rate of fibre recruitment, firing rate and motor unit synchronization. SIT in conjunction with END training results in increased motor unit recruitment and total work performed during a repeated Wingate test compared to END training alone [54], which suggests that improvements in neuromuscular characteristics may partially explain the greater improvements in Wingate performance and anaerobic capacity following LV-HIT.

### Limitations and Future Directions

The sample size of experiment 1 (n = 6) was relatively small and it is therefore possible that a larger sample size may have yielded different results. However, while an increased sample size might have allowed for subtle differences between LV-HIT and END to be detected (for both glycogen depletion and intramuscular signalling), we do not believe that the limited statistical power associated with the small sample studies impaired our ability to conclude that both type I and IIA fibres are recruited during both of exercise protocols examined in the current study. Additionally, we have observed a dissociation between phosphorylation of AMPK and phosphorylation of ACC, an interesting result that reflects previously published results [18], [40], [55]. While we are unable to comment with certainty regarding why this dissociation exists, it is possible that differential phosphorylation of α1 and α2 AMPK sub-units [56], [57] may impair the ability to estimate changes in AMPK activity based on changes in global AMPK phosphorytion. Thus, while we were unable to conduct this analysis on samples from the present study, the potential that phosphorylation of ACC is a more sensitive measure of AMPK activity than phosphorylation of AMPK itself raises the possibility the fibre specific differences in ACC phosphorylation may be present following exercise. This represents an important area for future research. It would also be of interest to investigate whether similar acute activation of intramuscular signalling pathways following LV-HIT and END result similar activation and expression of PGC-1α and other genes associated with the induction of mitochondrial biogenesis in the hours following exercise.

### Perspectives/Conclusions

Numerous recent investigations have attempted to elucidate the mechanisms by which interval training and END training induce similar improvements in aerobic capacity and exercise performance (VO_2_peak, time to fatigue trails, etc.) [18], [19], [40], [55], [58]. Collectively, these studies suggest that the intramuscular signaling required to elicit these adaptations are equivalently stimulated by both low intensity exercise of long duration and high-intensity exercise of short duration. While the minimum duration of low intensity exercise required to elicit adaptation remains unknown, very brief bouts of high-intensity exercise appear to be sufficient [9], [12], [13]. From an exercise prescription perspective, exercise resulting in the largest degree of intramuscular signaling activation in the greatest amount of muscle fibres would presumably result in the greatest degree of adaptation. What remains largely unknown, however, is whether differences in fibre recruitment and the magnitude of signal activation within individual fibre-types between interval training and END exercise are obscured by whole muscle analyses. Our results demonstrate that LV-HIT and END produce similar: a) glycogen depletion across fibre types (indicative of similar fibre recruitment), b) changes in intramuscular signaling, and c) fibre-type specific increases in oxidative capacity, intramuscular glycogen and triglyceride content, whole-muscle capillary density and O_2_pulse. Remarkably, these results suggest that 30 minutes of END exercise at ∼65% VO_2_peak or 4 minutes of LV-HIT at ∼170% VO_2_peak induce comparable deviations of the intra-myocellular environment (glycogen content and signaling activation); correspondingly, training-induced adaptations resulting for these protocols, and other interval training and END protocols [1], [8], appear comparable. The one exception to this equivocality is the capacity of LV-HIT to induce greater improvements in anaerobic adaptations than END. Thus, our results suggest that the mechanisms responsible for adaptation following chronic LV-HIT and END are the same, with the supramaximal exercise intensity inherent with LV-HIT permitting their manifestation in significantly less exercise time.

Collectively, results from this study suggest that the potency of high-intensity exercise resides is its ability to activate the mechanisms that trigger the induction of exercise-induced adaptation in a drastically reduced exercise time compared to traditional END training. However, while END exercise at intensities greater than ∼65% VO_2_peak are of adequate intensity to elicit “maximal” fibre recruitment (i.e. type I and IIA fibres) and the manifestation of chronic adaptations, at present the minimal duration of exercise at these relatively low intensities required for comparable adaptations is not known. Our results, and others [9], suggest that 30 minutes is adequate; however, given that our assumptions regarding the minimal dose of exercise required for adaptations having been recently challenged [59], [60], the minimal duration of moderate intensity END required to induce adaptation is an interesting area for future research.
